# Increasing trends in incidence of preterm birth among 2.5 million newborns in Guangzhou, China, 2001 to 2016: an age-period-cohort analysis

**DOI:** 10.1186/s12889-020-09739-6

**Published:** 2020-11-04

**Authors:** Jinhua Lu, Dongmei Wei, Songying Shen, Xiaoyan Xia, Jianrong He, Yan Sun, Kin Bong Hubert Lam, Wei Bao, Huimin Xia, Xiu Qiu

**Affiliations:** 1 Division of Birth Cohort Study, Guangzhou Women and Children’s Medical Center, Guangzhou Medical University, Guangzhou, 510623 Guangdong China; 2 Provincial Key Clinical Specialty of Woman and Child Health, Guangzhou, Guangdong China; 3 Nuffield Department of Women’s & Reproductive Health, University of Oxford, Oxford, UK; 4 Nuffield Department of Population Health, University of Oxford, Oxford, UK; 5 Department of Epidemiology, College of public health, The University of Iowa, Iowa, USA; 6 Provincial Clinical Research Center for Child Health, Guangzhou, Guangdong China

**Keywords:** Epidemiology, Preterm birth, Age-period-cohort analysis, Secular trends, Parity

## Abstract

**Background:**

The incidence of preterm birth (PTB, < 37 weeks of gestation) has been increasing in China and many other countries in recent years. However, the causes of the increase were not well understood. The current study aims to examine the contribution of maternal age, period of delivery, and maternal birth cohorts to long-term trends in preterm birth in Guangzhou, China.

**Methods:**

In a retrospective population-based study, data were obtained from 2,535,000 singleton live births with 20–43 gestational weeks from 2001 to 2016 and recorded in the Guangzhou Perinatal Health Care and Delivery Surveillance System, in China. The age-period-cohort models were applied to investigate the temporal changes in incidences of PTB, stratified by parity.

**Results:**

The incidence of preterm birth steadily increased from 5.1% in 2001 to 5.9% in 2016, with larger rise in primiparous mothers (from 5.0 to 5.9%) compared to multiparous mothers (from 5.6 to 5.9%). A J-shaped and a V-shaped relationship were found between maternal age and PTB among primiparous and multiparous mothers, respectively. A linear cohort effect was found among primiparous mothers with the lowest risk of PTB [risk ratio (RR) = 0.81, 95% confidence interval (CI): 0.74 to 0.89] in 1961 and the highest risk (RR = 1.06, 95% CI: 1.00 to 1.13) in 1997 compared to the mothers born in 1981. An inverse U-shaped association between maternal birth cohort and PTB was found in multiparous mothers. There were weak decreasing period effects on the trend of overall PTB among multiparous mothers and on the trend of extremely (< 27 weeks) or very (28–31 weeks) PTB among both parity groups during the period of 2001–2012.

**Conclusions:**

Our findings showed the PTB incidences had been increasing in the past 16 years in Guangzhou, China and both maternal age and cohort effects contributed to these trends. Further studies are recommended on the impact of altered maternal age and parity on premature births and corresponding public education and public health policies.

**Supplementary Information:**

**Supplementary information** accompanies this paper at 10.1186/s12889-020-09739-6.

## Background

Preterm birth (PTB), defined as births before 37 completed gestational weeks, is an important global public health issue. It is estimated that 14.84 million newborns were born to preterm in 2014 worldwide, accounting for 10.6% of all live births [1]. Apart from being the leading cause of neonatal and under-5 child mortality worldwide [2], PTB also increases the risk of short-term and long-term morbidities, such as poor growth, acute morbidity, respiratory illnesses, neurocognitive disorders, and chronic disease in adulthood, which could lead to high social and economic burdens [3, 4].

Reasons for the rising trend of PTB around the world in recent years [5–7] remain elusive. Upward shift in maternal age, a known risk factor of PTB [8, 9], may partially explain the increasing trend in PTB [9, 10]. Maternal early life factors, such as childhood hardships [11] and being born in a household with lower socio-economic status [12, 13] have been associated with preterm delivery. A classic age-period-cohort analysis, simultaneously examining maternal age (age effect, reflecting biological changes and social processes) [14], delivery year (period effect, capturing change given a specific period such as advance in medical services, health policies), and the birth year of mothers (cohort effect, reflecting unique experience/exposure and contextual factors experienced by the cohort over their life time) [15], is therefore a useful tool to interpret trends of health outcomes [14, 16, 17]. Most previous studies examining the association of maternal age and delivering period with incidence of PTB did not take into account the role of maternal birth cohorts [5, 6, 18]. Only one study in the United States has explored the interaction among maternal age, period, and birth cohort and found a strong effect of maternal age and period on PTB and a weak effect of cohort among older African American mothers [11].

Maternal age at childbirth has been increasing and the distribution of parity has altered over the past decades in China due to the relaxation of the one-child policy [10, 12]. Recent studies have suggested higher risk of PTB in primiparas than that in multiparas among mothers with advanced age [13, 19], indicating that the changes in distribution of parity may modify the contribution of maternal age to PTB incidence at a population level. Understanding the mutual effects of parity and mothers’ childbearing age on longitudinal trends in PTB incidence is particularly important in areas where the distributions of such factors have changed remarkably. However, information on the effects of the changing PTB trends at a population level has been lacking.

The present study aimed to examine the contribution of these 3 time-dependent factors to the long-term trends in PTB from 2001 to 2016 in Guangzhou, China. We also assessed whether the complex effects were modified by parity.

## Methods

### Data sources and population

Data on all singleton live births (*n* = 2,553,803) at 20–43 gestational weeks from 2001 to 2016 were retrospectively obtained from the Guangzhou Perinatal Health Care and Delivery Surveillance System [7, 20] in Guangzhou, China. The data included maternal age, parity, delivery mode, gestational age at birth, newborn’s sex, and birth weight. We excluded mothers who were < 15 or ≥ 45 years of age (*n* = 1947), born either before 1961 or after 2000 (*n* = 411), non-Mainland Chinese (*n* = 8976) and with missing data on maternal age (*n* = 1881), parity (*n* = 2815), newborn’s sex (*n* = 2050) and birth weight (*n* = 723), resulting in 2,535,000 singleton live births in the present study. The study was approved by the institutional ethical committee board of Guangzhou Women and Children’s Medical Center (No.201924801).

### Age, period, and cohort

Maternal age at delivery (age) was grouped into seven intervals: 15–19, 20–23, 24–27, 28–31, 32–35, 36–39, and 40–44 years. Delivery years (period) were grouped into four 4-year categories (2001–2004, 2005–2008, 2009–2012, and 2013–2016). Ten 4-year maternal birth cohorts were obtained by subtracting age from period and labelled as the midpoint of the birth years (1961, 1965, 1969, 1973, 1977, 1981, 1985, 1989, 1993 and 1997). For example, those delivered during the period 2001–2004 at the age of 20–23 were born between 1978 and 1984, thus the midpoint, 1981, was used to represent their birth cohort.

### Outcome measurements

Gestational age was confirmed based on an ultrasound examination in the first or second trimester [21]. When the examination was unavailable, gestational age was calculated according to the last menstrual period. PTB was defined as delivery before 37 gestational weeks.

### Statistical analysis

Poisson regression models with robust variance were used to estimate the change per year of PTB incidence and 95% confidence intervals (CIs). We also examined the interaction effects between maternal delivery year and parity to test whether the change of PTB incidences among primipara and multipara differed over the 16-year period using Poisson regression model. Age-specific PTB incidences were plotted using heatmaps by maternal delivery period and birth cohort for both parity, respectively.

The age-period-cohort (APC) analysis models were used to investigate the effects of maternal age, delivery year and birth year of mothers on the change trends of PTB incidence. The method described by Carstensen was used to solve the exact linear dependency (i.e., *age = period – cohort*) of 3 time-related factors [22]. The effect of the “drift” variable [22–24] (a combination of linear period effects and cohort effects) was estimated for the overall linear trend of PTB incidence, which cannot be attributable uniquely to period or cohort effects. Deviations from linearity (termed curvature), which are uniquely attributable to period or cohort effects and not dependent on any model constraint, were then estimated as period and cohort effects. To detect non-linear effects on PTB incidence trend, we fitted the parametric smooth functions based on natural splines with 3–6 knots for age, period, and cohort. The full models were carried out as a priori and submodels sequentially were fitted with age by adding drift, period and cohort variables. The deviance of the models was used to measure the goodness of fit. The statistical significance of the difference in deviances between the subsequent models was assessed by a log-likelihood ratio test. A smaller deviance value of the model indicates a better goodness fit to the data.

We performed a sensitivity analysis by restricting to births before 32 completed gestational weeks to explore the influence of the 3 time-related factors on extremely (< 27 weeks) or very (28–31 weeks) preterm delivery, which is an important indicator of improvement of modern intensive care for the premature infant.

The age-period-cohort models were performed separately by primiparas and multiparas, using the apc.fit function in the Epi package [25] in R (version 3.5.2). The period of 2001–2004 and the year of 1981 (the median maternal birth year among PTB cases) were chosen as the reference groups.

## Results

### Trends in PTB incidence

**Fig. 1:**
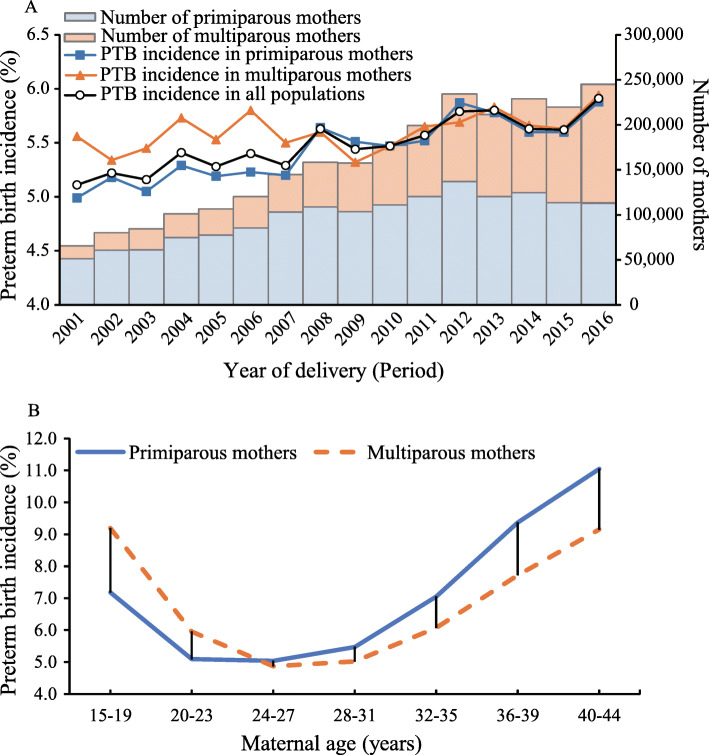
Temporal trends for preterm birth incidence by parity in Guangzhou, China, 2001–2016

A total of 2,535,000 mothers with singleton live births were included in the final analysis, of which 1,565,992 (61.8%) were primiparous and 969,008 (38.2%) were multiparous. Figure 1a illustrated the incidence of PTB in the full population and in subgroups of parity from 2001 to 2016 in Guangzhou, China. The overall incidence steadily increased from 5.1% in 2001 to 5.9% in 2016 with an average annual increase of 8.2‰ [95% confidence interval (CI), 6.9‰ to 9.4‰], among which the incidence increased from 5.0% in 2001 to 5.9% in 2016 (annual increase: 9.7‰, 95%CI, 8.1‰ to 11.4‰) in primiparous mothers and from 5.6 to 5.9% in multiparous mothers (annual increase: 4.5‰, 95%CI, 2.3‰ to 6.6‰). Significant interaction between maternal delivery years and parity on trend of PTB was observed (*P* < 0.001). Before 2008, the incidence of PTB among multiparous mothers was consistently higher than that among primiparous mothers. After that, the incidences among these two groups became similar. Figure 1b showed the different gap of PTB incidences between the two parity groups on each maternal age. The incidences of PTB among primiparous mothers with age ≥ 28 were higher than multiparous mothers, while opposite results in women who were younger than 23 years old. The maternal and birth characteristics of all mothers and by parity were presented in Supplementary Table 1 [see Additional file 1].

### Maternal age specific trends by year of delivery (period) and birth cohort

**Fig. 2:**
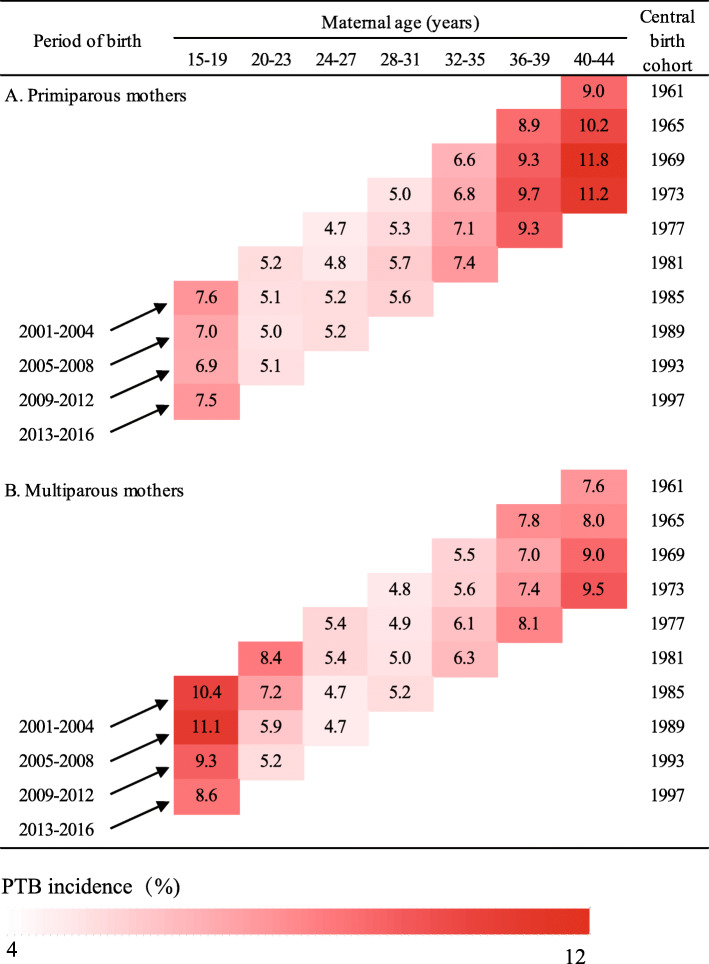
Incidences of preterm birth by maternal age, period, and birth cohorts in Guangzhou, China, 2001–2016

Figure 2 revealed that each generation experienced higher incidences than the generation preceding it among primiparous mothers after the age of 24. For instance, among the mothers with age of 40–44, the incidence of PTB rose from 9.0% in mothers born in 1961 to 11.2% in mothers born in 1973. The same was also observed among multiparous mothers older than 28 years. However, an opposite trend was found for younger (age of 15–27) multiparous mothers, who were born in more recent cohorts.

### Age-period-cohort effects on preterm birth

Supplementary Table 2 [see Additional file 1] summarized the results from the fit of the APC models by parity for PTB incidence, in which age, period, and cohort year were modeled continuously with splines. The change in residual deviance in the sequence of model building showed that age-drift and age-cohort models obviously improved the fitness over the age-only model for primipara and age-cohort and the full APC models were better than the age-only model for multipara.

**Fig. 3:**
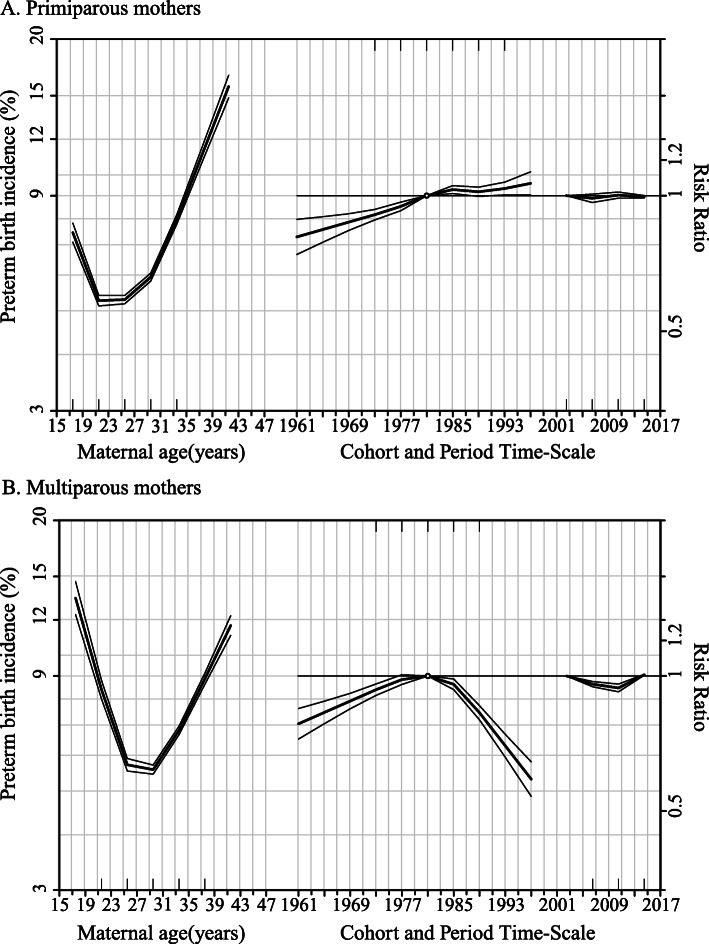
Age-period-cohort influences on trends in preterm birth by parity: Guangzhou, China, 2001 to 2016. The left curve showed the fitted age-specific incidence at the reference cohort (1981), the middle curve was the risk ratios of cohorts relative to the reference cohort (1981), and the right curve was the risk ratios of period conditional on the estimated age and cohort effects

Figure 3 showed the age-period-cohort effects on preterm delivery stratified by parity. The maternal age effect (the left curves) showed the change trend of preterm delivery incidence across different maternal age among two different parity groups were generally similar, where incidences of PTB were decreasing at younger age and then remained constant but were increasing at advanced age. However, age effects displayed a J-shaped curve among primiparous mothers and a V-shaped curve in multiparous mothers, where the nadir of preterm birth incidence appeared earlier in primiparous mothers (approximately 22 to 26 years) than in multiparous mothers (approximately 25 to 30 years).

Cohort and period effects reported as risk ratios of PTB are the curves in the middle and on the right of Fig. 3a and b, respectively. When compared to the mothers born in the median year of 1981, a gradually growing risk ratio of PTB, from 0.81 (95%CI, 0.74 to 0.89) in 1961 to 1.06 (95%CI, 1.00 to 1.13) in 1997, was observed among primiparous mothers born between 1961 and 1997 whereas risk ratio in multiparous mothers increased before 1981 but fell later.

The risk ratios of preterm delivery in primiparous mothers remained stable across the study period when compared to the reference period of 2001–2004. For multiparous mothers, there was a slight decline in risk ratios of PTB in the period of 2001–2012 (Risk ratio, 2005–2008: 0.96, 95%CI, 0.95 to 0.97; 2009–2012: 0.94, 95%CI, 0.92 to 0.96) compared with the period of 2001–2004.

When restricting to extremely or very PTB, the age and the cohort effects were similar to those for overall PTB. The period effects showed a V-shaped curve among both parity, where the nadir of risk ratios appeared in 2009–2012 (Risk ratio, primiparous mothers: 0.89, 95%CI, 0.84 to 0.93; multiparous mothers: 0.88, 95%CI, 0.83 to 0.93). The findings were presented in Supplementary Fig. 1 and Supplementary Table 3 (see Additional file 1).

## Discussion

In a large sample of 2.5 million pregnant women from Guangzhou, China, we observed that the PTB incidence increased linearly from 2001 to 2016. We also found there were strong effect of maternal age, moderate effect of birth cohort and no or weak effect of delivery period on PTB. A J-shaped and a V-shaped relationship were found between maternal age and PTB among primiparous and multiparous mothers, respectively. Among first delivery mothers, the incidence of PTB was higher among those born in more recent birth cohorts than those born earlier whereas an inverse U-shaped association between maternal birth cohort and risk of PTB was found in multiparous mothers. We observed that weak decreasing period effects on the trend of PTB among multiparous mothers during the period of 2001–2012 and that the risks of extremely or very PTB among both groups in 2009–2012 were the lowest compared with other periods.

Our study builds on previous studies regarding the association of maternal age and delivering period with the incidence of PTB, and further explored the effect of birth cohort and the modification effect by parity. To our knowledge, there has been only one study examining the maternal age-period-cohort effect on PTB in the American population [11]. While our finding on the association between maternal age and PTB is in agreement with that observed in the US study, Ananth and colleagues found that year of delivery had strong effects on PTB and weak effects for mother’s birth cohort [11], which was different from our findings. Interestingly, different patterns of maternal age and maternal birth cohort effect were observed between primiparous and multiparous mothers in the present study, which had not been explored in previous related studies. A comprehensive understanding of interaction effects of maternal age, delivery period and maternal birth cohort on PTB and modification effects of parity should inform the design and implementation of public health interventions.

Consistent with previous study [8] both younger and older maternal age increased the risks of PTB regardless of parity. The increasing trend in preterm birth was mainly driven by upward shift in maternal childbearing age due to experiencing high levels of economic and mental stress, especially in primiparous mothers [26]. Also, the proportion of advanced-age of multiparous mothers has risen owing to change of family child planning policy, [12] contributing to the increasing trend of PTB. Our study also showed that primiparous mothers who had higher risk of PTB were younger than multiparous mothers in older age. Larger increase in the prevalence of chronic conditions by maternal age in primipara than multipara [13, 27] may explain, at least partly, the trend towards younger maternal age with PTB among primipara.

Our study showed that lower risks of extremely or very PTB among both primiparous and multiparous mothers who delivered their children in the period around 2009–2012. Increasing level of educational attainment, enhancement of perinatal care service provision and technological advance in PTB prevention over the past decade may have contributed to the decreasing trend of extremely or very PTB. In our population, we found higher education level was associated with lower risk of preterm delivery, especially among primiparous mothers (Data not shown). However, other risk factors of PTB, such as health problems triggered by urbanization and increasing use of assisted reproductive technology, may counteract the effects of improvement of medical care services, which may partially explain the effects of periods of extremely or very PTB tended to be null in the more recent years [28, 29]. In Guangzhou, we found higher incidence of preterm birth in the central area than that in the suburban area over time (Data not shown).

Our findings showed that mothers born in more recent era had a higher risk of PTB among primiparous mothers as well as among multiparous mothers who born before 1981. Mothers born in more recent birth cohort experience remarkable lifestyle change, increased environmental pollution, and higher stress due to rapid change of economy and society, which may pose profound impacts on women’s reproductive system [30–32]. For example, prevalence of obesity in both children and adult female increased rapidly over past two decades in China [28, 33]. Evidence has shown that obesity is associated with inflammatory up-regulation and higher risk of metabolic disorders, such as insulin resistance and lipotoxicity, which may disturb the function of reproductive system during their lifetime and ultimately increase the risk of PTB [34]. However, a decreased cohort effect was also observed among multiparous mothers who born after 1981 but not in primiparous mothers.

We observed that the effects of maternal age or maternal birth cohorts on PTB differed by parity. Although the mechanisms are still not fully understood, it might be partly due to the difference in maternal health conditions, socio-economic background and psychological pressure and so on at each age group between primiparous and multiparous mothers. Further studies are warranted to determine which factors are relevant to our observed effects of maternal age and birth cohorts on PTB and how they might interact with each other.

Although a large sample size included in the analysis enabled us to estimate robust results, several limitations should be considered in the study. Firstly, data on some explanatory variables, such as cigarette smoking, a known risk factor of PTB, were not available in the electronic surveillance database of birth registration; thus we were unable to elucidate the detailed mechanisms of observed maternal age and birth cohort on PTB in this study. However, it is worth noting that in China, the prevalence of cigarette smoking among pregnant women is lower than 1% [35, 36]. Secondly, although it is currently standard practice to confirm gestational age using ultrasound examination at first or early second trimester in Guangzhou [21], the last menstrual period based method was also used in some rural areas, especially during the early delivery period, which might have resulted in misclassification of PTB. Thirdly, although age-period-cohort models can provide an exploratory descriptive tool for examining population health patterns, they cannot establish causal inference of disease incidence. Fourthly, the data in our study was from a single city in China, which might limit the generalization of the results. However, Guangzhou is a mega city with a large population (14.5 million) [37] which is comparable to or even larger than the nationwide population size of many countries around the world.

From a public health perspective, this study suggests that young and advanced-age mothers have higher risk of PTB, and primiparous mothers at higher risk of PTB were younger than multiparous mothers in older age. Thus, prenatal care and interventions should be targeted at young and older pregnant women, particularly for primiparous mothers. Since the implementation of universal 2-child policy in January 2016, there has been a substantial rise in the number of advanced-age pregnant women and premature infants. Allocation of resources for reversing the rising trend of PTB and preventing and treating PTB-related complications in children are warranted. Besides, as Guangzhou is one of the most developed cities in China, our study will provide evidence for the future change of PTB in other developing regions.

## Conclusions

Our findings showed the PTB incidences had been increasing in the past 16 years in the Guangzhou, China and both maternal age and cohort effects contributed to these trends. With constant social-economic growth and implementation of universal 2-child policy in China, more attention on the impact of altered maternal age and parity on premature births and corresponding public education and public health policies is needed in the future.

## Supplementary Information

**Additional file 1.** Supplemental Tables 1–3 and Supplemental Figs. 1. Supplementary Table 1 Maternal and newborn characteristics by parity among singleton live births in. Guangzhou, China from 2001 to 2016. Supplementary Table 2 Analysis of deviance for Age-Period-Cohort models for preterm birth incidence, stratified by parity. Supplementary Table 3 Analysis of deviance for Age-Period-Cohort models for extremely or very preterm birth incidence, stratified by parity. Supplementary Fig. 1 Age-period-cohort influences on trends in extremely or very preterm birth by parity in Guangzhou, China, from 2001 to 2016

## Data Availability

The raw data was obtained from the Guangzhou Perinatal Health Care and Delivery Surveillance System. These data are not publicly available due to the restricted policy of the institution. Data may be available upon request to the Guangzhou Women and Children’s Health Information Center, given researchers and project protocols meet the criteria and have obtained an approval for the data access (enquiry email: Weidong.Li@gwcmc.org).

## Abbreviations

PTB: Preterm birth
RR: Risk ratio
CI: Confidence interval
APC: Age-period-cohort
