# Supplementary material for: Increasing trends in incidence of preterm birth among 2.5 million newborns in Guangzhou, China, 2001 to 2016: an age-period-cohort analysis
Source: BMC Public Health. 2020 Nov 4;20:1653. doi: 10.1186/s12889-020-09739-6 (PMC7640687; doi:10.1186/s12889-020-09739-6)
Supplement: Supplementary file 1 — Additional file 1. Supplemental Tables 1–3 and Supplemental Figs. 1. Supplementary Table 1 Maternal and newborn characteristics by parity among singleton live births in. Guangzhou, China from 2001 to 2016. Supplementary Table 2 Analysis of deviance for Age-Period-Cohort models for preterm birth incidence, stratified by parity. Supplementary Table 3 Analysis of deviance for Age-Period-Cohort models for extremely or very preterm birth incidence, stratified by parity. Supplementary Fig. 1 Age-period-cohort influences on trends in extremely or very preterm birth by parity in Guangzhou, China, from 2001 to 2016 [file 12889_2020_9739_MOESM1_ESM.pdf]

Supplementary Table 1 Maternal and newborn characteristics by parity among singleton live births in Guangzhou, China from 2001 to 2016

Characteristics	All mothers		Primiparous mothers		Multiparous mothers	
	N	%	N	%	N	%
Gestation age of birth (weeks)						
20-31	12,863	0.5	7226	0.5	5637	0.6
32-34	33,167	1.3	19,671	1.2	13,496	1.4
35-36	94,974	3.8	59,239	3.8	35,735	3.7
⩾37	2,393,996	94.4	1,479,856	94.5	914,140	94.3
Maternal age (years)						
15-19	73,908	2.9	67,163	4.3	6745	0.7
20-23	464,914	18.3	379,990	24.3	84,924	8.8
24-27	828,230	32.7	583,480	37.3	244,750	25.3
28-31	698,326	27.6	386,939	24.7	311,387	32.1
32-35	323,845	12.8	113,408	7.2	210,437	21.7
36-39	117,581	4.6	29,078	1.8	88,503	9.1
40-44	28,196	1.1	5934	0.4	22,262	2.3
Central birth cohort (year)						
1961	1005	0.0	398	0.0	607	0.1
1965	11,243	0.4	4352	0.3	6891	0.7
1969	59,906	2.4	23,893	1.5	36,013	3.7
1973	210,013	8.3	99,342	6.3	110,671	11.4
1977	397,173	15.7	223,259	14.3	173,914	17.9
1981	597,747	23.6	359,770	23.0	237,977	24.6
1985	627,824	24.8	403,415	25.8	224,409	23.1
1989	448,517	17.7	311,007	19.9	137,510	14.2
1993	155,741	6.1	118,241	7.5	37,500	3.9
1997	25,831	1.0	22,315	1.4	3516	0.4
Delivery modes						
Vaginal delivery	1,551,045	61.2	929,981	59.4	621,064	64.1
Cesarean delivery	983,955	38.8	636,011	40.6	347,944	35.9
Newborn's sex						
Male	1,370,660	54.1	818,379	52.3	552,281	57.0
Female	1,164,340	45.9	747,613	47.7	416,727	43.0

Supplementary Table 2 Analysis of deviance for Age-Period-Cohort models for preterm birth incidence, stratified by parity

Models[a]	Change in Residual Deviance (Degrees of Freedom) [b]	
	Primipara	Multipara
Age		
Age-drift[c]	68.3 (1)***	0 (1)
Age-Cohort	26.1 (4)***	154.9 (3)***
Age-Period-Cohort	3.1 (2)	36.9 (1)***
Age-Period	-24.5 (-4)***	-186.7 (-3)***
Age-drift[c]	-4.7 (-2)	-5 (-1)*

[a]Models are ordered so that adjacent rows provide tests between models, culminating in the age-period-cohort model.

[b]Changes in residual degree of freedom and deviance are used to perform a χ^2 test between adjacent models, where the fuller model is accepted if the test is significant.

[c]The age-drift model is the intersection of the age–period and the age–cohort models.

*P<0.05

***P<0.001

Supplementary Table 3 Analysis of deviance for Age-Period-Cohort models for extremely or very preterm birth incidence, stratified by parity

Models[a]	Change in Residual Deviance (Degrees of Freedom) [b]	
	Primipara	Multipara
Age		
Age-drift	58.4 (1)***	17.5 (1) ***
Age-Cohort	33.1 (4) ***	44.4 (3) ***
Age-Period-Cohort	30.1 (2) ***	19.6 (1) ***
Age-Period	-43.4 (-4) ***	-57.2 (-3) ***
Age-drift	-19.8 (-2) ***	-6.8 (-1) **

[a]Models are ordered so that adjacent rows provide tests between models, culminating in the age-period-cohort model.

[b]Changes in residual degree of freedom and deviance are used to perform a χ^2 test between adjacent models, where the fuller model is accepted if the test is significant.

**P<0.01

***P<0.001

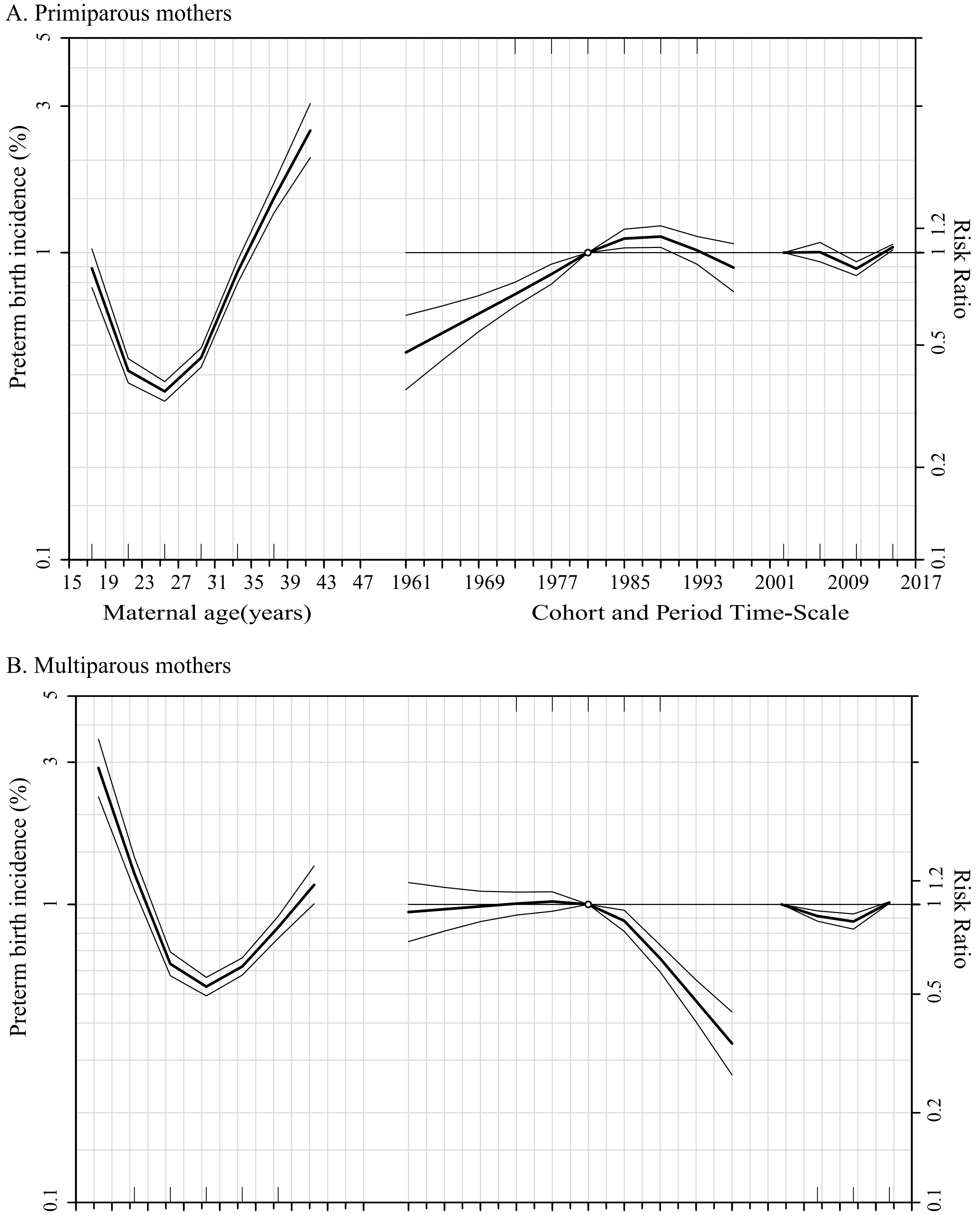

Supplementary Fig.1 Age-period-cohort influences on trends in extremely or very preterm birth by parity in Guangzhou, China, from 2001 to 2016.

The left curve showed the fitted age-specific incidence at the reference cohort (1981), the middle curve was the risk ratios of cohorts relative to the reference cohort (1981), and the right curve was the risk ratios of period conditional on the estimated age and cohort effects.
